# Primary care physicians’ approaches to low-value prescribing in older adults: a qualitative study

**DOI:** 10.1186/s12877-022-02829-7

**Published:** 2022-02-24

**Authors:** Aimee N. Pickering, Eric L. Walter, Alicia Dawdani, Alison Decker, Megan E. Hamm, Walid F. Gellad, Thomas R. Radomski

**Affiliations:** 1 Division of General Internal Medicine, University of Pittsburgh School of Medicine, Pittsburgh, PA USA; 2 Center for Health Equity Research and Promotion, VA Pittsburgh Healthcare System, Pittsburgh, PA USA; 3 Present Address: Center for Research on Healthcare, 3609 Forbes Avenue, 2nd Floor, Pittsburgh, PA 15213 USA; 4 Department of Medicine, University of Pittsburgh Medical Center, Pittsburgh, PA USA; 5 Center for Pharmaceutical Policy and Prescribing, Health Policy Institute, University of Pittsburgh, Pittsburgh, PA USA

**Keywords:** Low-value care, Low-value prescribing, Medication value, Deprescribing, Polypharmacy

## Abstract

**Background:**

Low-value prescribing may result in adverse patient outcomes and increased medical expenditures. Clinicians’ baseline strategies for navigating patient encounters involving low-value prescribing remain poorly understood, making it challenging to develop acceptable deprescribing interventions. Our objective was to characterize primary care physicians’ (PCPs) approaches to reduce low-value prescribing in older adults through qualitative analysis of clinical scenarios.

**Methods:**

As part of an overarching qualitative study on low-value prescribing, we presented two clinical scenarios involving potential low-value prescribing during semi-structured interviews of 16 academic and community PCPs from general internal medicine, family medicine and geriatrics who care for patients aged greater than or equal to 65. We conducted a qualitative analysis of their responses to identify salient themes related to their approaches to prescribing, deprescribing, and meeting patients’ expectations surrounding low-value prescribing.

**Results:**

We identified three key themes. First, when deprescribing, PCPs were motivated by their desire to mitigate patient harms and follow medication safety and deprescribing guidelines. Second, PCPs emphasized good communication with patients when navigating patient encounters related to low-value prescribing; and third, while physicians emphasized the importance of shared decision-making, they prioritized patients’ well-being over satisfying their expectations.

**Conclusions:**

When presented with real-life clinical scenarios, PCPs in our cohort sought to reduce low-value prescribing in a guideline-concordant fashion while maintaining good communication with their patients. This was driven primarily by a desire to minimize the potential for harm. This suggests that barriers other than clinician knowledge may be driving ongoing use of low-value medications in clinical practice.

## Introduction

Low-value prescribing (LVP), defined as the use of medications whose costs or harms exceeds their potential benefits, is common in older adults and results in inappropriate medication use and polypharmacy (the use of ≥5 medications) [1]. An estimated 30–50% of community-dwelling adults aged 65 or older have been prescribed at least one potentially inappropriate medication or have been subject to polypharmacy, which places them at risk for adverse drug events, hospitalizations and unnecessary medical expenditures [2–4].

LVP also results in significant wasteful spending at the health system level, with an estimated $14.4–29.1 billion spent annually in the U.S. on potentially low-value medications [4–6]. In response, health systems and payers have increasingly implemented interventions to reduce LVP [7]. Primary care providers (PCPs) are often faced with decisions regarding LVP. As such, many interventions, such as educational initiatives or decision-support tools within the electronic medical record, target PCP decision making and aim to support them in deprescribing potentially low-value medications [7], yet PCPs’ strategies for navigating patient encounters and meeting expectations surrounding LVP have not been well characterized [8, 9]. This makes the development and implementation of acceptable interventions to reduce LVP challenging. Thus, the objective of this study was to characterize primary care physicians’ (PCPs) perspectives on and approaches to reduce LVP in older adults through qualitative analysis of clinical scenarios discussed during semi-structured interviews.

## Methods

### Study design and sample

During September–October 2019, we conducted semi-structured interviews of PCPs who care for patients aged ≥65 years as part of a larger study examining PCPs’ perspectives on LVP. We chose semi-structured interviews as our method of qualitative data collection to best characterize PCPs’ individual perspectives, especially ones that they may be hesitant to share in a larger group setting. We recruited PCPs within the University of Pittsburgh Medical Center (UPMC), which is a large not-for-profit integrated health care system with 40 hospitals and 700 outpatient practices in Pennsylvania, New York and Maryland. PCPs were recruited via a general solicitation email and were eligible to participate if they reported providing primary care to patients aged ≥65 years and practiced for at least one half-day per week. We chose to only include PCPs in this study as they are often faced with clinical encounters involving LVP. PCPs are well-positioned to make decisions regarding LVP as they have access to the patient’s full medical history to help inform decisions around medications, often have an established relationship with patients that supports shared decision-making and have the ability to follow patients longitudinally [8]. We did not include subspecialists (e.g., cardiologists or surgeons) as they may only be able to comment on certain classes of medications. We sought to recruit 15–20 PCPs trained in various specialties (i.e. family medicine, general internal medicine, geriatrics) and from varied practice settings (i.e. academic medical centers vs community outpatient clinics) in order to capture diverse approaches to low-value prescribing. Academic medical centers are affiliated with a medical school and are the primary sites for graduate medical education and federally funded research whereas community outpatient clinics are not as involved in graduate medical education or research. It has been previously demonstrated that prescribing practices differ between these two settings [10, 11]. Our recruitment goal was based on qualitative research guidelines to achieve thematic saturation [12].

The study protocol was reviewed and approved by the University of Pittsburgh Institutional Review Board. All methods were carried out in accordance with relevant guidelines and regulations. The content in the following sections is informed by the Consolidated Criteria for Reporting Qualitative Research (COREQ) reporting guidelines [13]. Informed consent was obtained at the start of the interviews for all participants.

### Data collection

Two clinical scenarios involving potential LVP were presented as part of a semi-structured interview. The clinical scenarios were discussed for approximately 10–15 minutes during the 30–40 minutes semi-structured interviews which also addressed PCPs’ broad views on medication value, incentives to participate in or reduce low-value prescribing and the acceptability of system-level interventions to reduce low-value prescribing, the results of which are presented elsewhere [14]. We chose to analyze and present the PCP responses to clinical scenarios separately in order to specifically identify themes related to how PCPs approach LVP during patient encounters rather than combining these themes with more generalized views on medication value and LVP. The clinical scenarios and corresponding interview guide were developed by members of the research team, which was comprised of practicing general internists, in addition to experts in healthcare value, pharmacoepidemiology and qualitative analysis. The scenarios were based upon real-life clinical encounters and pilot tested by a general internist prior to the start of the study. They involved two common scenarios involving LVP: (1) Deprescribing potentially low-value medications, and (2) a patient’s request to begin a new potentially low-value medication. Full scenarios are shown in Table 1. We specifically asked PCPs to describe their approaches to prescribing, deprescribing, and meeting patient expectations surrounding LVP within the context of these scenarios. Interviews were conducted by telephone by a research assistant experienced in qualitative interview techniques (Dawdani). We chose to utilize a research assistant as the interviewer, rather than another member of the research team such as a physician or health services researcher to limit potential bias related to personal experiences with patients. The interviewer met the participants for the first time on the day of the interview and thus had no preexisting relationship with participants prior to the start of the interviews. Participants had no knowledge of the interviewer outside of her role within the study team (i.e. no knowledge of personal goals or motivations). Interviews were audio-recorded and transcribed verbatim. There were no other non-participants present at the time of the interviews.

**Table 1:** Clinical Scenario Interview Prompts

Scenario	Prompt
Scenario #1: Deprescribing	Ms. A is an eighty-one-year-old woman with a history of hypertension and hyperlipidemia. She is a lifelong nonsmoker and engages in thirty minutes of moderate physical activity three days a week. Her blood pressure is 118/72, pulse 68 bpm, and body mass index is 28. She’s currently prescribed aspirin 81 mg po daily, Atorvastatin 20 mg daily, Lisinopril 10 mg daily, Carvedilol 12.5 mg bid, Pantoprazole 40 mg daily, Docusate 100 mg bid and takes a Calcium and Vitamin D supplement. The patient reports that she is comfortable on her current medications and does not experience any side effects or financial hardships as a result.
Scenario #2: Prescribing new potentially low-value medication	Mr. S is a sixty-eight-year-old man with a history of hypertension, hyperlipidemia and well-controlled type II diabetes mellitus. You’re seeing him for a fifteen-minute follow-up visit where he complains of fatigue and erectile dysfunction. He continues to have a desire to engage in sexual intercourse but is unable to maintain an erection. He requests a prescription for a testosterone supplement as a friend has similar symptoms and found that testosterone was helpful and improved his overall quality of life.

### Codebook development and data analysis

We conducted thematic analysis according to the multistep process outlined by Braun & Clark [15]. Two members of the research team (Walter, Decker) familiarized themselves with the transcribed data and then organized it by assigning relevant tags to segments of the transcribed data through a process known as “coding.” A qualitative codebook was generated from the content of 12 transcripts using an iterative, inductive approach known as “editing.” In this approach, codes are generated from the content of the transcripts with minimal inference from a specific theory-based framework or what might be expected in the literature [16]. The codebook was further refined by the principal investigator prior to use (Radomski). The same members of the research team then independently applied the codebook to each transcript, after which they met to reconcile discrepancies in coding. One member of the research team (Pickering) and the principal investigator (Radomski) then used the organized data (i.e., the coding) to conduct a final thematic analysis to identify salient themes within the data [15, 17]. The themes were reviewed by a third member of the research team (Walter) as a form of investigator triangulation [18]. Three investigators – 1) a health services researcher and general internist, 2) a clinical research fellow and general internist, and 3) a general internal medicine resident were involved in the final thematic analysis in order to confirm that they saw the same themes within the data. Thematic saturation, defined as consistency and redundancy of perspectives, was achieved.

## Results

We interviewed 16 PCPs (11 general internal medicine, 2 family medicine, and 3 geriatrics) from 6 unique outpatient practices. A majority of participants (*n* = 10) were female and all were non-Hispanic white. Participants had been practicing medicine for a median of 15 years post-residency with a median of 5 clinical half-days per week. Nearly all (*n* = 14) had appointments as clinical medical school faculty.

When presented with the first scenario, all PCPs stated they would deprescribe at least 2 out of 7 of the medications and 14 of the 16 PCPs would deprescribe at least half of the medications. Aspirin (15/16 PCPs), pantoprazole (14/16), and docusate (14/16) were the most frequently cited due to their potential for harm or lack of potential benefit. One PCP captured the opinions of the majority of clinicians who were interviewed, stating “I would think she’s on a lot more than she needs to be and I could probably get that down to maybe one blood pressure medicine and her statin.” When presented with the second scenario, all clinicians considered testosterone supplementation to be of low-value due to lack of evidence and potential for adverse effects and would not prescribe this medication despite the patient’s request.

We identified 3 key themes across both scenarios regarding physicians’ clinical decision making related to LVP. Additional representative quotes are shown in Table 2. First, when deprescribing, *physicians were motivated by their desire to mitigate patient harms and to follow medication safety and deprescribing guidelines and thus prioritized medications with the greatest potential for adverse drug events followed by those with lack of potential benefit.* In the first scenario, most PCPs prioritized aspirin and pantoprazole for deprescribing due to potential for adverse effects such as bleeding and increased risk of pneumonia. They also weighed the potential benefit that the older patient in the scenario would have from medications such as statins and anti-hypertensives, often referencing current medication guidelines, when considering which medications to discontinue. One PCP highlighted this theme when she stated, “… the aspirin I would deprescribe because there is actual harm to being on it which is the bleeding risk and I don’t perceive a significant benefit.” Similarly, another PCP considered the risks of anti-hypertensives, stating “I’d be a little bit cautious in an eighty-one-year-old not to have her blood pressure too much lower than what it already is…. Carvedilol same you know caution regarding blood pressure but also perhaps some caution regarding heart rate.” Another PCP was influenced by medication guidelines when considering the potential benefit of atorvastatin, stating “I’m not sure what age cut off the most recent study that actually looked at this population I believe over the age of seventy-five that does show very limited benefit… for primary prevention in coronary artery disease so I would probably stop that as well.”

**Table 2:** Key Themes and Supplemental Quotes

Theme	Representative Quote
When deprescribing, physicians were motivated by their desire to mitigate patient harm and to follow medication safety and deprescribing guidelines and thus prioritized medications with the greatest potential for adverse drug events followed by those with lack of potential benefit	“I’d probably prioritize the pantoprazole because it has more potential for adverse effects. The docusate isn’t going to help but I’m not aware of a lot of specific adverse effects to her.” “I’m not sure there is anything in her history that warrants her being on aspirin. It may just increase her risk of… bleeds without conveying much reduction in risk of coronary artery disease or stroke.” “…so I would be asking ‘Does she have an indication for this? And if not, can we get rid of it?’…so I would say without an indication that is definitively a low-value medication.”
Physicians emphasized the importance of good communication with patients regarding decisions about low-value medications	“I don’t want to feel like I’m putting up barriers to their medical care or their complaint’s not being taken seriously… sometimes in that situation though I just advise that they get a second opinion [from] a specialist.” “I think what I… would do as a primary care doc is not prescribe testosterone and really not even pursue a diagnosis of low testosterone but perhaps refer [him] to a urologist who specializes in erectile dysfunction and see what alternatives they might offer him.” “I would probably ask her especially for the pantoprazole and the docusate… if she’d ever tried coming off of them… and explore with the patient and share decision making what her thoughts were about whether or not she really needed them or if she was willing to try to come off of them.”
Physicians ultimately prioritized patients’ well-being over satisfying their expectations to begin or remain on a low-value mediation with the potential for harm	“I’m not going to prescribe a low-value medication just because a patient has requested it, I just think that we need to talk more about it so we can get on the same page.” “But when I feel strongly that something… has harms then I guess I am less likely to take the patient preference” “…the patient really shouldn’t force your pen to the prescribing pad”

Second, *physicians emphasized the importance of good communication with patients regarding decisions about low-value prescribing.* They were often willing to order additional tests and seek the opinions of other clinicians to gather additional data to better explain their decision making to patients and to make patients feel that their concerns about their health were being addressed. In the second scenario, many PCPs stated they would order testosterone levels in order to more effectively communicate with the patient about their decision not to prescribe testosterone supplementation. For example, one PCP stated she would obtain additional blood work because she “owe[s] the patient… more of an in-depth explanation for why I’m not giving it to him.” Similarly, another PCP stated “…it might make me pursue testing that I may not otherwise have pursued in order to have more data in that conversation with a patient, so if we check the testosterone and it is normal or not very low, then that gives me additional reasons for explaining why it is low value.” Other PCPs stated they would refer to specialists to demonstrate that the patient’s concerns were being addressed. One PCP stated “So I try not to make it adversarial if we’re kind of on opposite sides I try… and facilitate like a second opinion for them but if it is kind of feeling like the risks outweigh the benefits from my perspective, I won’t go forward with it but I will try to kind of honor their request and get them set up for kind of further evaluation or a second opinion with another doctor.”

Third, *physicians ultimately prioritized patients’ well-being over satisfying their expectations to begin or remain on a low-value mediation with the potential for harm*. In both scenarios, clinicians were willing to diverge from a patient’s preferences and make recommendations to reduce the potential for harm that stemmed from a patients’ use or desire to use potentially low-value medications. For example, in the second scenario, one PCP stated they would not prescribe testosterone despite the patient’s request because “testosterone… can potentially carry serious risks, like cardiovascular disease, sleep apnea, polycythemia… so it comes along with potentially some, some very kind of serious issues….” Another PCP highlighted this theme when he stated “I try to do what I think is best and not let the patient influence me in doing things that I don’t think are appropriate or of value.” Another PCP described how she frames conversations where patient preference does not align with her judgement, stating “I usually frame this as just telling the patients ‘It’s my job to only do things that I think are more beneficial than risk for you.’”

## Discussion

Among a cohort of primary care physicians who care for older adults, we determined that they prioritize deprescribing medications that are most likely to be harmful followed by those that are minimally effective. They also valued good communication with patients but ultimately prioritized patients’ well-being over their preferences to start or remain on a potentially low-value medication.

Several prior qualitative studies have examined clinicians’ perspectives on deprescribing in the primary care setting. This prior work identified similar themes with regards to weighing risks versus benefits and building a trusting relationship with patients when making decisions around deprescribing. For example, D’Avanzo et al. found that factors such as health risks related to polypharmacy were the main reasons general practitioners would deprescribe. They also found the need for effective communication with patients and caregivers so that patients did not feel practitioners were “giving up on them” when deprescribing medications [19]. Similarly, Anderson et al. found that general practitioners and clinical pharmacists weigh harms and benefits when making deprescribing decisions and that a continuous therapeutic relationship with patients was essential to deprescribing [20]. These studies also examined barriers to deprescribing such as patient expectations or other system factors such as fragmented care or influence from specialists. We did not specifically ask PCPs about barriers to deprescribing in our clinical vignettes but instead focused on their baseline approaches to encounters involving low-value prescribing.

Additionally, in a survey-based study examining general practitioners’ views on deprescribing in adults aged greater than 80, Jungo et al. presented a clinical vignette involving a frail adult on 7 long-term medications. They found that factors such as “risk and benefits of medications,” “patients’ quality of life,” and “patients’ life expectancy” to influence their deprescribing decisions [21]. This aligns with our findings that PCPs consider a medication’s potential for harm and lack of potential benefit when deprescribing. In contrast to our study, the authors also found “patients’ fear of potential negative health outcomes from deprescribing” to influence their decisions around deprescribing. While the PCPs in our study reported that they would consider a patient’s preferences, they ultimately prioritized a patient’s well-being when deprescribing. This difference may be explained, in part, by the fact that we conducted semi-structured interviews that did not probe specifically for PCPs’ thoughts on patients’ fears around deprescribing whereas this survey asked participants to rank this factor. Our work builds upon these prior studies by presenting clinical scenarios during semi-structured interviews, which allowed us to illicit themes around clinical-decision making beyond those factors on a survey. Additionally, while prior studies largely focused on perspectives related to deprescribing, we also identified PCP views on starting a new potentially low-value medication.

When presented with clinical scenarios outside of the constraints of real-life clinical encounters, physicians in our cohort generally adhered to medication safety and deprescribing guidelines [22–27]. In a prior study examining PCPs’ broad views on medication value, incentives to participate in or reduce low-value prescribing and the acceptability of system-level interventions to reduce low-value prescribing, we demonstrated that while PCPs wish to avoid LVP, they find it difficult to address in real life practice due to a variety of factors, including perceived patient expectations, deference to subspecialists, time constraints and misaligned quality metrics and incentives [14]. This suggests that ongoing use of low-value medications and polypharmacy is driven by these perceived barriers, rather than a lack of knowledge about potentially LVP in older adults.

Interventions that align with PCPs’ perspectives and baseline approaches to reduce LVP may be more readily accepted and implemented in clinical practices. In fact, our themes correspond with many of the domains within with the Theoretical Domains Framework (TDF). This validated theoretical framework includes 14 domains that affect behavior and has been widely used in the implementation of healthcare interventions [28, 29]. For example, many PCPs in our study were motivated by their *social/professional role and identity* to prioritize well-being and similarly by their *beliefs about consequences* that may result from low-value prescribing. Aligning interventions with these domains may result in greater behavior change around low-value prescribing. Such interventions should also target system-level barriers in a way that is acceptable to clinicians rather than focusing on provider knowledge base. For example, a decision-support tool within the electronic medical record that flags high-risk medications may both decrease the perceived cognitive and time burden associated with deprescribing while also aligning with a clinician’s prioritization of harmful medications. Although not the focus of the current study, examining barriers to LVP within the context of TDF would be another avenue to develop interventions that are more readily adopted and sustained in clinical practice.

It is especially important that interventions bridge the perception gap between prescribers and patients with regard to LVP. Prescribers often cite patient expectations and reluctance to participate in deprescribing as a barrier to reducing low-value medication use, yet prior studies have shown that patients are often amenable to deprescribing [30–32]. The perspectives of PCPs in our study mirror those of patients who cite factors such as effectiveness, adverse effects on quality of life, and relationship with prescriber as influencing their perceived value of a medication [33]. Interventions that prompt clinicians and patients to participate in shared-decision making surrounding LVP may empower both parties to recognize their shared values and decrease the perceived burden placed on clinicians.

A key limitation of the study is that our sample was homogenous in terms of race/ethnicity and level of training (i.e. all non-Hispanic white physicians) and the majority practiced in academic settings. Therefore, our findings may not generalize to PCPs with different levels of training or of more diverse backgrounds. Our cohort also included only 16 PCPs; however, the high degree of concordance in their responses indicates thematic saturation. Lastly, because we presented idealized scenarios, there is risk of social desirability bias, and PCP responses may not fully reflect clinician decision making in real-life patient encounters. However, the use of individual semi-structured interviews allowed PCPs to share potentially sensitive views and perspectives.

## Conclusion

In conclusion, our findings highlight the importance of understanding physicians’ approaches to LVP and deprescribing in the context of common clinical scenarios. Taken together, they may allow health systems and payers to more effectively develop policies and interventions that align with these perspectives and bridge the gap between clinicians’ and patients’ views on LVP.

## Data Availability

The datasets generated and analyzed during the current study period are not publicly available in order to maintain participant privacy and confidentiality but are available from Dr. Radomski (email: radomskitr@upmc.edu) on reasonable request.

## Abbrevations

PCP: primary care physician
LVP: low-value prescribing
